# Team-Based Learning in Prosthodontics Courses: Students' Satisfaction

**DOI:** 10.1155/2022/4546381

**Published:** 2022-01-20

**Authors:** Selma A Saadaldin, Elzahraa Eldwakhly, Sundus Naji Alaziz, Alhanoof Aldegheishem, Amal M El sawy, Maha M. Fahmy, Sahar M. Alsamady, Nozha M. Sawan, Mai Soliman

**Affiliations:** ^1^Prosthodontics Division, Schulich School of Medicine and Dentistry, Western University, London, Canada; ^2^Department of Clinical Dental Sciences, College of Dentistry, Princess Nourah bint Abdulrahman University, P. O. Box 84428, Riyadh 11671, Saudi Arabia; ^3^Mathematical Science Department, College of Science, Princess Nourah bint Abdulrahman University, Riyadh, Saudi Arabia; ^4^Department of Preventive Dental Sciences, College of Dentistry, Princess Nourah bint Abdulrahman University, P. O. Box 84428, Riyadh 11671, Saudi Arabia

## Abstract

The goal of this cross-sectional observational study was to assess dental students' satisfaction regarding team-based learning (TBL) methodology in prosthodontics courses taught at College of Dentistry, Princess Nourah bint Abdulrahman University, Saudi Arabia. Undergraduate dental students at second, third, fourth, and fifth years were taught prosthodontics courses through traditional and TBL pedagogies. TBL sessions consisted of preparation, readiness assurance, and application. At the end of each prosthodontics course, the students were asked to complete a self-administered questionnaire that was divided into four sections to assess the effect of TBL on the following parameters: information acquisition, interpersonal skills improvement, classroom environment, and the students-instructors interaction. The responses of the questionnaire followed the Likert scoring method (scaled from 1 to 5). The *t*-test and ANOVA statistical analyses were performed using SPSS. *Results*. The response rate to the questionnaire was 86%. There were a significant relationship and correlation between TBL pedagogy and student satisfaction (*P* values ≤ 0.05) for all levels. The means of the responses for the second and fifth years were 4.36 and 4.56, respectively, where the means for the third and fourth years were 3.54 and 3.59, respectively. The parameter notably affected by TBL was interpersonal skills enhancement. All students strongly agreed that TBL enhances personal flexibility and boosts their self-esteem. *Conclusion*. Students showed positive perceptions about TBL pedagogy in terms of active engagement, knowledge acquisition, and improvement of interpersonal skills leading to more efficient learning outcome.

## 1. Introduction

Team-based learning (TBL) shifted the learning pendulum from passive faculty-centered learning to active student-centered learning. Larry Michaelsen originated TBL in the 1970s [1]. Traditional education centers on the educators, where the students' role in didactic lecture-based education is limited to listening, understanding, and retaining the information. With the team-based learning, learning responsibility falls on students to prepare beforehand and participate actively in the classroom. TBL develops a mature, confident class-participation and interaction and enhances students' critical thinking, team-work, and communication skills [2, 3] These skills contribute to improve clinical performance [4, 5].

TBL is used extensively in several countries across the world for healthcare education of physicians, dentists, nurses, and other health care professionals [6]. TBL process encompasses three phases: preparation, readiness assurance, and course objective application. In the preparation phase, reading assignments are appointed to students to independently read and study the assigned material before class time. The readiness assurance phase is divided into two parts. During the first part of the second phase, multiple-choice questions (MCQ) exam is taken by students individually to confirm their readiness to apply their self-learned knowledge gained during the preparation phase. Students retake the same MCQ exam as a team of 6-7 students during the second part. In the course objective application phase, teams complete in-class application assignments that are based on collaboration, knowledge use, and deficiencies identification [1, 3, 5, 7–9].

Many studies proved that dental students do not reach the predetermined educational goals through the traditional dental school curricula [10]. Continuous assessment of the existing educational state is a necessary process to identify its strengths and flaws in order to reach proper clinical education curricula [7, 8]. Surveying and evaluating the student's opinions across their teaching experience are reliable methods for evaluating the quality of the educational process [9–11]. Several studies showed positive perceptions on TBL, where the students become better problem-solvers, where they enjoyed the TBL interactive environments that led to more knowledge recalling [5, 12–14]. Students described TBL as an encouraging proactive peer-to-peer learning method [15], whereas in other studies the students rated TBL learning process as moderate [16], and other students became anxious and frustrated and were little interested in TBL due to the lack of traditional lectures and placing the learning responsibilities on them [4, 13].

Our study aims to assess dental students' satisfaction regarding TBL pedagogical methodology in prosthodontics courses taught at the College of Dentistry, Princess Nourah bint Abdulrahman University (PNU), Riyadh, Saudi Arabia. The current study's null hypothesis stated that there is no effect of the TBL on the student satisfaction on the following parameters: knowledge acquisition, interpersonal skills improvement, classroom environment, and the students-instructors interaction.

## 2. Materials and Methods

Ethical approval of the study (IRB approval 19-0155) was obtained from the ethics committee of the Deanship of Research at PNU, to conduct a cross-sectional descriptive survey study. The prosthodontics division launched TBL pedagogy to teach part of preclinical and clinical prosthodontics courses for the second (D2), third (D3), fourth (D4), and fifth (D5) years at the College of Dentistry, Princess Nourah bint Abdulrahman University in Saudi Arabia. Prosthodontic faculty members attended a TBL workshop before applying the TBL methodology, and they facilitated the sessions.

The TBL sessions were either three-hours (3h-TBL) that were taught for D2 and D5 or one-hour (1 h-TBL) that were taught to D3 and D4 because D3 and D4 have tight schedules that are mixed between clinical and sim-clinical sessions, so the allocated time for didactic was 1 hour only; therefore, researchers of the current study decided to modify the TBL session for D3 and D4 to compare between the traditional TBL sessions that usually last for hours and modified TBL that is 1 hour only. For each year five sessions were taught using TBL. The subjects were as follows: interim removable partial dentures, denture bases, and basics of removable partial denture design for D2. Provisional restorations, cementation, and management and treatment of traumatized oral tissues for D3. Laminate veneers, management of residual ridge resorption, and fixed prosthodontics risk management for D4. For D5, the topics of TBL were advanced removable partial denture design, geriatric dentistry, and advance restorative dentistry.

Dental students were divided into 6 teams, each composed of 5–7 students. The materials of the TBL were sent to the students one week before the sessions. At the beginning of each session, students took short MCQ quizzes individually (iRAT) and then retook the same quizzes as a team within their assigned groups (tRAT).  Supplementary Materials shows sample of the quizzes for D3. A discussion session followed out to review the questions and provide the student with feedback regarding their answers. Later, clinical cases were discussed amongst the groups. At the end of the last TBL sessions, students completed a self-administered questionnaire regarding their TBL sessions' experiences. Students' answers would be anonymous and confidential. The questionnaire was adapted from the previous study [17] where it had 20 statements measured by a five-point Likert scoring method, ranging from one (strongly disagree) to five (strongly agree). The four sections of the questionnaire assessed the effect of TBL on knowledge acquisition, interpersonal skills improvement, classroom environment, and the students-instructors interaction. The Cronbach alpha coefficient measured the reliability of the questionnaire. The values of the coefficient were 0.963–0.974, indicating that the questionnaire has internal consistency. SPSS (version 20.0; SPSS, Chicago, IL, USA) was used to analyze the collected data using *t*-test and ANOVA statistical analyses. *P* values ≤ 0.05 indicated statistical significance.

## 3. Results and Discussion

### 3.1. Results

86% of the dental students (135 out of 156) filled the questionnaire. The percentages of the participant student from each year were as follows: D2 (95%), D3 (81%), D4 (100%), and D5 (69%). Students' age ranged from 19 to 23 years. Calculations for the question's scores were as follows: strongly agree 4.20–5, agree 4.19–3.40, neutral 3.39–2.60, disagree 2.59–1.8, and strongly disagree 1.79–1. The four parameters of the questionnaire were knowledge acquisition, interpersonal skills improvement, classroom environment, and the students-instructors interaction.

Table 1 displays the average scores for each statement in the first parameter (knowledge acquisition). The statement that had the highest score with D2 was “It helps to memorize things for a longer time” while the statement that had the highest score with D3 and D4 students was “It boosts students' contribution to learning in the class.” The statement that scored the highest with D5 was “TBL helps to learn more in the class.”

Table 2 shows the average scores for each statement in the second parameter (interpersonal skills improvement). D2 had the highest score for the statement “It helps to grow the reasoning and problem-solving abilities.” While the statement that had the highest score for D3 and D4 was “It encourages team-work.” The statement “it helps to develop critical thinking” had the highest score for D5. The third parameter was the classroom environment. The statement “It creates more opportunities for questions and answers in the class” had the highest score for D2, though the statement “It improves classmates' interactions” had the highest score with D3, D4, and D5.

Table 3 shows average scores for each statement in the third parameter (class environment). D2 and D5 strongly agreed with all statements of the parameter while D3 and D4 responses were ranged between agree and neutral.

The statement “It helps to create a more convenient interaction with the teacher” had the highest score for all levels of the last parameter as shown in Table 4, which disclose the students-instructors relationship. D2 and D5 responses were similar for all parameters where the students strongly agree with the majority of the statements. Likewise, a similarity existed between third and fourth year responses that also agree with most of the statements.

Table 5 compares the mean of the responses of the four dental levels within the four parameters, and the average of the responses was agree for all parameters. The one-way ANOVA test showed a significant relationship between all questionnaire parameters and the students' satisfaction where *P* values ≤ 0.05. The Pearson correlation coefficient showed a strong and significant relationship between the parameter variables within each level and within the four levels, Pearson correlation coefficient >0.5.

### 3.2. Discussion

The purpose of the study was to evaluate dental students' satisfaction regarding TBL sessions in prosthodontic courses. The study's null hypothesis was rejected, and there is significant effect of the TBL on the student satisfaction with all parameters mentioned in the study: knowledge acquisition, interpersonal skills improvement, classroom environment, and the students-instructors interaction.

Health science educators verified that TBL inspire, engage, and motivate students to acquire knowledge compared to the traditional teaching methodology that does not provoke students' learning skills [5, 17–20]. Students who participated in the current study agreed that TBL sessions assisted them to learn more in the class and to memorize the information for a longer time, and it kept them motivated for studying and learning as well as it boosted their contribution in class as well as boosting students' contribution to learning in the class. The substantial students' satisfaction was consistent with other studies [11, 14, 15, 21–23]. In these studies, students preferred TBL to other traditional learning methods because they enjoyed the active and interactive learning approach that led to deeper understanding and a better engagement level. Interestingly, 3 h TBL sessions had higher satisfaction scores than 1 h TBL sessions. This can be related to having more time for in-class discussion and the facilitators' constructive feedback. Students need adequate time to become accustomed to TBL as they are involved in the learning process, and they should be effective, active engaged learners [4]. Carbrey et al. recommended that students completing the MCQs (iRAT) at home individually allow more time for tRAT and group discussion that need a higher level of thinking [19].

Repeated testing through iRAT and tRAT during TBL sessions support retrieval of new knowledge and helps in knowledge recall [18]. Students reported a consensus agreement at the current study for the first parameters' statements, specifically that TBL helped to memorize information and helped to learn more in the class. Studies have shown that TBL predominantly promotes learner-to-learner engagement and improves interpersonal skills. Researchers proved that most engagements in TBL were learner-to-learner as opposed to learner-to-facilitator or learner-to-self [24, 25]. Students appreciated the need for increased individual accountability for learning and identified value in learning through discussion, both characteristics inherent to team learning and deep and critical thinking that are needed for dental students. Students are concerned about their team grade through tRAT which is dependent on group performance [26]. This concern encourages friendly competition between the teams [7] which is considered as one of the TBL strengths [27]. The team will benefit from various factors such as different personalities, learning attitude, former knowledge, and topic interest which will lead to the best learning outcomes for the students [25]. The majority of the students in the current study agree that TBL helped to develop critical thinking and encouraged teamwork which are crucial qualities in the dental career.

Attributes of a passive lecturer who merely transfers information are different from those of an active TBL facilitator who organizes and conducts a student-focused TBL activity with a variety of facilitating skills to promote effective learning. Therefore, this study's instructional design factors should be considered when designing and implementing TBL courses to improve overall student satisfaction. TBL improve the class environment efficiently. Students in the current study agreed that TBL makes a more pleasing atmosphere at the class as there will be more time to exchange the information between the students and more opportunities for discussion and asking questions. Students agreed that TBL encouraged classmates to be more punctual and improved classmates' interaction. All these positive and high levels of satisfaction of the current study's TBL sessions can be accredited to the efforts exerted by both facilitators and students toward TBL sessions as a new teaching modality applied in the prosthodontics courses to relieve the usual anxiety and stress associated with the courses [28]. The most useful interaction reported was the student-instructor interaction that expands the students' comfort and understanding zones.

Prosthodontics courses in dentistry are considered challenging and stressful subject that are considered one of the dental curriculum's main components that requires a high level of skill, preparation, and planning [28]. The implementation of TBL in prosthodontics courses proved to enhance students' performance, which reflected in greater student engagement with less demand on faculty members' contribution. Students reported 72% full satisfaction rates for TBL incorporation in the teaching strategy of preclinical prosthodontics courses [29]. The majority of dental students in the current study reported positive responses to acquiring a deeper understanding of the prosthodontics courses' academic content accompanied with self-directed learning willingness. TBL highly elaborated the students' initiative to independent learning, informed acceptance of responsibility for one's own learning, creativity, and the ability to use basic study and problem-solving skills [30].

Limitations of the study are as follows: the data represented dental students' satisfaction at the prosthodontic department, and this information should be checked with other dental disciplines. Students at Princess Nourah bint Abdulrahman University are only female, so the reaction of different gender is missing.

## 4. Conclusions

Students at Princess Nourah bint Abdulrahman University were highly satisfied regarding application of the TBL methodology in the prosthodontics course. Longer sessions (3 hours) received more satisfaction than the short sessions (1 hour) as the students have more time for engagement and discussion. Facilitators had more time to provide the students with their feedback regarding the MCQs and the clinical cases. This study supports the future application of TBL in dental curricula especially in the demanding courses.

## Figures and Tables

**Table 1:** Mean, standard deviation, and level of statements of the first parameter (knowledge acquisition), according to the Likert scoring method where SA is referred to strong agree, A is referred to agree, and N is referred to neutral.

Statements/mean (SD)/level	D2	D3	D4	D5
TBL helps to learn more in the class	4.41 (0.93) SA	3.55 (1.24) A	3.33 (1.16) N	4.66 (0.61) SA
It helps to memorize things for a longer time	4.47 (0.71) SA	3.24 (1.09) A	3.62 (1.14) A	4.48 (0.74) SA
It creates more motivation for study and learning	4.18 (0.87) A	3.45 (1.33) A	3.59 (1.10) A	4.55 (0.74) SA
It boosts students' contribution to learning in the class	4.18 (0.87) A	3.85 (0.85) A	3.82 (0.85) A	4.48 (0.79) A
It helps to get through deep-reading and learning	4.06 (0.74) A	3.4 (1.29) A	3.49 (0.94) A	4.34 (0.86) SA

**Table 2:** Mean, standard deviation, and level of statements of the second parameter (interpersonal skills improvement) according to the Likert scoring method where SA is referred to strong agree and A is referred to agree.

Statements/mean (SD)/level	D2	D3	D4	D5
It helps to grow the reasoning and problem-solving abilities	4.50 (0.56) SA	3.52 (1.12) A	3.67 (1.3) A	4.66 (0.61) SA
It enriches interpersonal skills	4.29 (0.87) SA	3.75 (1.08) A	3.51 (1.05) A	4.55 (0.69) SA
It helps to develop critical thinking	4.41 (0.82) SA	3.90 (1.08) A	3.77 (1.06) A	4.72 (0.60) SA
It enhances personal flexibility and being respectful to others	4.26 (0.90) SA	3.90 (1.08) A	3.67 (0.96) A	4.52 (0.74) SA
It increases self-esteem	4.18 (1.30) SA	3.9 (1.08) A	3.49 (0.94) A	4.52 (0.79) SA
It encourages teamwork	4.47 (0.75) SA	4 (1.10) A	3.8 (1.06) A	4.62 (0.68) SA

**Table 3:** Mean, standard deviation, and level of statements of the third parameter (class environment), according to the Likert scoring method where SA is referred to strong agree, A is referred to agree, and N is referred to neutral.

Statements/mean (SD)/level	D2	D3	D4	D5
It creates more opportunities for questions and answers in the class	4.44 (0.71) SA	3.38 (1.37) N	3.56 (1.12) A	4.62 (0.68) SA
It makes a more pleasing atmosphere at the class	4.29 (0.76) SA	3.66 (1.14) A	3.62 (1.02) A	4.55 (0.74) SA
It causes a better use of classroom time	4.21 (0.81) SA	3.10 (1.32) N	3.56 (1.17) A	4.48 (0.88) SA
It increases the students' attention at the class	4.38 (0.60) SA	3.31 (1.37) N	3.59 (1.09) A	4.48 (0.83) SA
It encourages classmates to be more on-time and punctual	4.12 (0.88) SA	3.66 (1.26) A	3.64 (1.04) A	4.24 (0.87) SA
It improves classmates' interactions	4.35 (0.69) SA	3.86 (1.37) A	3.77 (1.09) A	4.66 (0.61) SA
It causes better recognition of the classmates' abilities	4.41 (0.56) SA	3.69 (1.26) A	3.67 (0.96) A	4.48 (0.74) SA

**Table 4:** Mean, standard deviation, and level of statements of the fourth parameter (students-instructors interaction), according to the Likert scoring method where SA is referred to strong agree, A is referred to agree, and N is referred to neutral.

Statements/mean (SD)/level	D2	D3	D4	D5
It helps to understand the teacher's morale and concerns	4.47 (0.67) SA	3.38 (1.27) N	3.41 (1.14) A	4.62 (0.68) SA
It helps to create a more convenient interaction with the teacher	4.56 (0.56) SA	3.41 (1.21) A	3.64 (1.14) A	4.66 (0.614) SA

**Table 5:** Comparison of the mean (standard deviation) of the four parameters for D2, D3, D3, and D4 levels, according to the Likert scoring method where SA is referred to strong agree and A is referred to agree.

	D2	D3	D4	D5	Mean
First parameter: knowledge acquisition	4.25 (0.55) SA	3.49 (0.92) A	3.57 (0.89) A	4.51(0.66) SA	3.95 A
Second parameter: interpersonal skills improvement	4.36 (0.61) SA	3.80 (0.87) A	3.65 (0.88) A	4.59 (0.60) SA	4.09 A
Third parameter: classroom environment	4.32 (0.51) SA	3.5 (0.99) A	3.63 (0.94) A	4.5 (0.61) SA	3.98 A
Fourth parameter: students-instructors interaction	4.51 (0.52) SA	3.39 (1.14) A	3.53 (1.08) A	4.66 (0.53) SA	4.02 A
Mean	4.36 SA	3.54 A	3.59 A	4.56 SA	

## Data Availability

The data used to support the findings of this study are available from the corresponding author upon request.

## Supplementary Materials

Supplementary Materials Sample of the MCQ quiz for the TBL session for D3 that was attempted by the students individually (iRAT) and then attempted as a team within the assigned students groups (tRAT).
