# Supplementary material for: Team-Based Learning in Prosthodontics Courses: Students' Satisfaction
Source: Int J Dent. 2022 Jan 20;2022:4546381. doi: 10.1155/2022/4546381 (PMC8794685; doi:10.1155/2022/4546381)
Supplement: Supplementary Materials — Sample of the MCQ quiz for the TBL session for D3 that was attempted by the students individually (iRAT) and then attempted as a team within the assigned students groups (tRAT). [file 4546381.f1.docx]

**International Journal of Dentistry**

**Team-Based Learning in Prosthodontics Courses; Students' Satisfaction**

**Appendix 1**: Sample of MCQ quiz for TBL session for D3 that done by the students individually (iRAT) and then done as a team within the assigned students groups (tRAT).
